# Focused and Radial Shock Wave Therapy in the Treatment of Tennis Elbow: A Pilot Randomised Controlled Study

**DOI:** 10.1515/hukin-2015-0068

**Published:** 2015-10-14

**Authors:** Piotr Król, Andrzej Franek, Jacek Durmała, Edward Błaszczak, Krzysztof Ficek, Barbara Król, Ewa Detko, Bartosz Wnuk, Lidia Białek, Jakub Taradaj

**Affiliations:** 1 The Jerzy Kukuczka Academy of Physical Education, Department of Physiotherapy Basics, Katowice. Poland.; 2 Medical University of Silesia, School of Medicine, Chair and Department of Medical Biophysics, Katowice. Poland.; 3 Medical University of Silesia, School of Health Sciences, Chair Department of Rehabilitation, Katowice. Poland.; 4 Department of Physical Culture and Health Promotion, University of Szczecin, Szczecin. Poland.

**Keywords:** extracorporeal shock wave therapy, lateral epicondylitis, treatment

## Abstract

The purpose of this article was to evaluate and compare the efficacy of radial and focused shock wave therapies applied to treat tennis elbow. Patients with tennis elbow were randomized into two comparative groups: focused shock wave therapy (FSWT; n=25) and radial shock wave therapy (RSWT; n=25). Subjects in the FSWT and RSWT groups were applied with a focused shock wave (3 sessions, 2000 shocks, 4 Hz, 0.2 mJ/mm^2^) and a radial shock wave (3 sessions, 2000 + 2000 shocks, 8 Hz, 2.5 bar), respectively. The primary study endpoints were pain relief and functional improvement (muscle strength) one week after therapy. The secondary endpoint consisted of the results of the follow-up observation (3, 6 and 12 weeks after the study). Successive measurements showed that the amount of pain patients felt decreased in both groups. At the same time grip strength as well as strength of wrist extensors and flexors of the affected extremity improved significantly. Both focused and radial shock wave therapies can comparably and gradually reduce pain in subjects with tennis elbow. This process is accompanied by steadily improved strength of the affected extremity.

## Introduction

Tennis elbow is a painful condition affecting the outside part of the elbow, which is associated with observable tenderness of the lateral epicondylus of the humeral bone. Resistant extension of the wrist and fingers and supination of the forearm clearly increase the amount of pain that frequently radiates peripherally. The tennis elbow syndrome seriously disturbs the function of the upper extremity (Shiri and Viikari-Jantura, 2011).

The condition is frequently difficult to treat. The two approaches that are employed to this end are conservative treatment and surgery (Shiri and Viikari-Jantura, 2011; Johnson et al., 2007). The first approach makes use of physiotherapy (Shiri and Viikari-Jantura, 2011; Johnson et al., 2007; Trudel et al., 2004), special orthoses (Johnson et al., 2007; Oken et al., 2008), pharmacotherapy (Johnson et al., 2007; Trudel et al., 2004), autologous blood injection (Johnson et al., 2007) and acupuncture (Johnson et al., 2007; Trudel et al., 2004; Trinh et al., 2004).

Physiotherapeutic treatment modalities include eccentric resistance and stretching exercises (Stasinopoulos et al., 2005), Mills’ manipulation (Stasinopoulos et al., 2004) and deep friction massage (Trudel et al., 2004; Stasinopoulos et al., 2004), as well as ultrasound (Trudel et al., 2004; Oken et al., 2008), ultraphonophoresis (Trudel et al., 2004), ionophoresis (Trudel et al., 2004) and laser radiation (Trudel et al., 2004; Oken et al., 2008).

Focused Shock Wave Therapy (FSWT) has been used for conservative treatment of tennis elbow for over 20 years now (Thiel, 2001). The number of original studies available on this subject is considerable, but optimal parameters of FSWT ensuring its effectiveness are yet to be determined (Haake et al., 2002; Hammer et al., 2000; Krischek et al., 1999; Speed et al., 2002). The world history of Radial Shock Wave Therapy (RSWT) is much shorter. The number of original reports on its efficacy in the treatment of various conditions is steadily rising, however, it is still insufficient to enable the determination of the optimal application variables of a shock wave (Avancini-Dobrovic et al., 2011; Gerdesmeyer et al., 2008; Gunduz et al., 2012; Spacca et al., 2005). Studies comparing the efficacy of both modalities applied to the same condition are even more rare (Lohrer et al., 2010; Van der Worp et al., 2014).

A prospective randomised study was designed to assess and compare the efficacy of focused and radial extracorporeal shock wave therapies applied to treat tennis elbow. The primary study endpoints were pain relief and functional (muscle strength) improvement one week after therapy. The secondary endpoint consisted of the results of the follow-up observation (3, 6 and 12 weeks after the treatment).

## Material and Methods

Ethical approval for the study was granted by the Local Research Ethics Committee (reference No KNW/0022/KB1/158/10). All participants gave their written informed consent to participate.

### Participants

The patient inclusion criteria were as follows: (1) painful tennis elbow during at least 3 previous months; (2) painful palpation of the lateral epicondyle; (3) painful resisted middle finger and wrist extension (Thomson test).

Patients meeting the following criteria were excluded from the experiment: (1) younger than 18 years; (2) local infection; (3) malignancy; (4) bilateral tennis elbow; (5) carpal tunnel syndrome; (6) medial epicondylitis; (7) elbow arthritis or instability; (8) generalised polyarthritis; (9) ipsilateral shoulder dysfunction; (10) neurological abnormalities; (11) radial-nerve entrapment; (12) cardiac arrhythmia or a pacemaker; (13) cancer; (14) diabetes; (15) physical therapy and/or a corticosteroid injection administered within the previous six weeks; (17) pregnancy.

Patients who consented to participate in the study were randomly allocated in a one-to-one ratio to either the FSWT group or the RSWT group. Before the study commenced, its coordinator, who was not involved in patient selection and inclusion, delivered non-transparent and sealed envelopes containing patient’s treatment allocation. Using a computerised number generator, the study statistician gave random numbers to the envelopes. After final enrolment and baseline assessment, the study researcher allocated sequential numbers to participants and handed them the envelope with the corresponding number. The envelopes were opened when individual participants met with the treating clinician. The study researcher in charge of the follow-up measurements was blinded to treatment allocation for the length of the trial.

The FSWT group consisted of 25 patients (13 men and 12 women) with a mean age of 47.64 ±7.62 years, a mean body mass of 76.40 ±17.15 kg and a mean height of 171.0 ±7.69 cm. The right upper extremity was affected in 21 patients and the left upper extremity in 4. The dominant and non-dominant extremities represented 22 and 3 cases, respectively. The average duration of the condition was 8.44 ±7.52 months. All patients in this group were treated with a focused shock wave.

The RSWT group included 25 patients (11 men and 14 women) with a mean age of 45.76 ±7.52 years, a mean body mass of 75.24 ±16.36 kg and a mean height of 170.36 ±6.86 cm). The right upper extremity was affected in 19 patients and the left upper extremity in 6. The dominant and non-dominant extremities represented 19 and 6 cases, respectively. The average duration of the condition was 7.16 ±6.28 months. All subjects included in this group were treated with a radial shock wave.

### Procedures

Focused shock wave therapy was administered without anaesthesia using the Wolf Piezowave device. The most sensitive point on patient’s lateral epicondyle received a total of 2000 shocks per session (4 Hz; 0.2 mJ/mm^2^). Each subject participated in 3 treatment sessions held at weekly intervals.

Radial shock wave therapy was also administered without anaesthesia and the device used was the Gymna Uniphy ShockMaster 500. First 2000 shocks (8 Hz, 2.5 bars) were applied to the most sensitive point of the lateral epicondyle and then 2000 shocks to the dorsal part of the forearm using the same frequency and pressure variables. Each patient participated in 3 treatment sessions held at weekly intervals.

### Measures

Before treatment and 1, 3, 6 and 12 weeks after its cessation, all patients in the FSWT and RSWT groups were assessed for the amount of pain they felt (rest pain, night pain and pain during activity). Strength of wrist flexors and extensors and grip strength of the affected and unaffected extremity were also established.

To measure the amount of pain the VAS scale was employed, where 0 indicates “no pain” and 10 indicates “most severe pain”.

Grip strength was measured using the Saehan dynamometer, model SH5001, in a subject sitting with the elbow joint flexed at 90°. Strength of wrist extensors was measured in the same position and using the same dynamometer, which was stabilised on the table. The moving arm of the dynamometer was always set in the lowest position. To measure strength of wrist extensors and flexors, subjects were asked to press the dynamometer with respectively the dorsal and palmar, distal part of the metacarpus (the force arm being the same for all participants). All subjects performed 3 maximum-effort trials separated by rest periods of 30 seconds. For statistical analysis average values from three measurements were used.

The variables that were analysed for each group of patients were the following:

- Average strength of wrist extensors and flexors in the affected and unaffected extremity, respectively;
- The ratio between average wrist extensor strength calculated for the affected and unaffected extremity;
- The ratio between average wrist flexor strength calculated for the affected and unaffected extremity;
- Average grip strength of the affected and unaffected extremity;
- The grip strength ratio between the affected and unaffected extremity.

Percentage change in muscle strength was derived from the following formula:

$$\text{X}[\%]=\frac{\left({\text{X}}_{\text{k}}-{\text{X}}_{\text{p}}\right)}{{\text{X}}_{\text{p}}}\times 100[\%]$$

where:

*X* – percentage change,
*Xp* – average strength before treatment,
*Xk* – average strength 12 weeks after treatment,

To calculate percentage change in the amount of pain felt by patients the following formula was used:

$$\text{X}[\%]=\frac{\left({\text{X}}_{\text{p}}-{\text{X}}_{\text{k}}\right)}{{\text{X}}_{\text{p}}}\times 100[\%]$$

where:

*X* – percentage change in the amount of pain,
*Xp* – average amount of pain before treatment,
*Xk* – average amount of pain 12 weeks after treatment.

### Statistical Analysis

According to the results of the Mann-Whitney U test conducted before the experiment, the comparative groups were homogenous in terms of all investigated variables.

The statistical analysis of changes recorded in the groups was performed using the post-hoc Tukey’s t-test. An assumption was made that differences were statistically significant at *p*≤0.05.

To make inter-group comparisons of changes, the Mann-Whitney U-test was adopted. The level of significance was set at *p*≤0.05.

## Results

Successive measurements showed that the amount of pain felt by patients in both comparative groups gradually decreased (Table 1).

Grip strength and strength of wrist extensors and flexors of the affected and unaffected extremity improved in both groups, the improvement being greater in the affected extremity (Table 2).

The ratios between wrist flexors, wrist extensors and grip strength of the affected and unaffected extremity steadily improved (with one exception) in both groups over the period of observation (Table 2).

Apart from strength of wrist flexors of the unaffected extremity, percentage changes in all analysed parameters were comparable between the groups (Table 3).

Apart from strength of wrist flexors of the unaffected extremity, percentage changes in all analysed parameters were comparable between the groups (Table 3).

## Discussion

According to the results of the study, extracorporeal shock wave therapy (ESWT) is a promising physical agent in tennis elbow treatment. Both early and long-term results show good tissue response. A significant relief in pain and significant improvement in function were noted, particularly during the follow-up observation.

A radial shock wave stimulates a much larger area of tissue than a focused shock wave. The effective focal zone of the latter is very small (Ogden et al., 2001), thus, the area of affected tissue that can be treated is also small. The radial shock wave allows treating the original site of the disease (e.g. the lateral epicondylar area), as well as other affected areas. The approach we adopted in the RSWT group differed from that proposed by other researchers managing subjects with ennis elbow and other conditions in that we treated both the lateral epicondylus and the dorsal part of the forearm (Cacchio et al., 2006; Gunduz et al., 2012; Ibrahim et al., 2010; Spacca et al., 2005).

In our experiment, all three types of pain decreased gradually and comparably between groups over the observation period. The reduction in pain did not differentiate the groups significantly.

In some experiments where researchers repeated the observation of subjects with tennis elbow, a shock wave (both focused and radial) was also found to reduce pain intensity over time (Ko et al., 2001; Lee et al., 2012; Rompe et al., 1996). An exception was one study where change in the pain level in patients did not significantly differentiate the treatment group from the placebo group (Speed et al., 2002).

Since patients with tennis elbow suffer from pain and decreased strength of the affected extremity, researchers argue that grip strength assessment can be a reliable indication of how efficacious the applied therapy has been (Snijders et al., 1987). Even so, not all researchers decide to measure strength of the affected extremity (Hammer et al., 2000; Ko et al., 2001; Melegati et al., 2004).

In our experiment, grip strength of the affected and unaffected extremity improved in both groups over the observation period, but steady progress was only noted for the affected extremity. It was observed that in both groups the grip strength ratio between the affected and unaffected extremity changed in favour of the affected side. This confirmed that grip strength of the affected extremity was coming back to normal in both groups.

Greater muscle strength after treatment was observed by other researchers as well (Benjamin et al., 1999; Gunduz et al., 2012; Spacca et al., 2005), excluding one experiment where after focused shock wave therapy it decreased (Rompe et al., 1996).

Only few researchers measured strength of wrist extensors and wrist flexors in their patients (Alizadehkhaiyat et al., 2007). The measurements we made before therapy and after each period in the follow-up period showed that the wrist flexors generated greater force than wrist extensors of the same extremity in both comparative groups (this pattern was noted for both affected and unaffected extremity). A similar correlation can be found in healthy subjects (Alizadehkhaiyat et al., 2007).

In the FSWT group, the ratios between strength of extensors and flexors of the affected and unaffected extremity were greater 12 weeks after treatment by 43.46% and 35.64%, respectively. In the RSWT group the increases were 58.86% and 23.03%. The improvements indicate that the affected extremity was recovering to its normal strength.

The reason for both extensors and flexors to increase in strength may have been weaker pain after treatment, allowing increased physical activity of the affected extremity. The fact that the flexor/extensor strength ratio did not improve significantly in the affected extremities although their strength in absolute terms was steadily improving can be attributed to physiological muscular balance between extremities before treatment. Although incorrect muscle balance is widely covered in research reports, there are no studies explaining the phenomenon with regard to forearm muscles in subjects with tennis elbow (Alizadehkhaiyat et al., 2007).

In the Benjamin et al.’s (1999) experiment involving patients with tennis elbow, strength of the wrist extensor of the affected extremity increased significantly, considerably more than in the unaffected arm, in patients who were subjected to a combination of physical therapies. In their experiment, pre-treatment strength of wrist flexors of the affected extremity accounted for 67.8% of that recorded for the unaffected arm, increasing after treatment to 95.2%.

There are no reports where focused and radial shock wave therapies applied to patients with tennis elbow are compared. We had found only two original studies that compared their effect on patients with other conditions.

Van der Worp et al. (2014) treated patients with patellar tendinitis. Forty three subjects (57 tendons) were randomized into two treatment groups (FSWT and RSWT). Both groups received three treatment sessions at 1-week intervals. All patients were delivered 2000 impulses per session (4 Hz, 0.12 mJ/mm^2^ (FSWT) and 8 Hz, 2.4 bar (RSWT), respectively). Moreover, the patients participated in an eccentric exercise programme that started 2 weeks after the last shock wave treatment. Follow-up measurements that were performed 1, 4, 7 and 14 weeks after treatment showed that FSWT and RSWT were not significantly different from each other regarding their efficacy. In both groups decreasing amounts of pain were observed and VISA-P questionnaires pointed to significant improvements in the subjects’ condition.

Lohrer et al. (2010) conducted a pilot study to compare the results of focused and radial ESWT applied to 39 patients with plantar fasciitis randomized into two groups. In both groups treatment was performed in three sessions. The shock wave parameters were 10 Hz, 2000 impulses, 3 bar (radial) and 0.2 mJ/mm^2^ (focused). The efficacy of the modalities was determined from a multivariate analysis of changes in the Foot Functional Index (FFI), neuromuscular performance (single leg drop and long jump, postural stability, isokinetic testing performed at different angular velocities) and a composite score from baseline to the 12 weeks’ follow up. The study provided some evidence that FSWT was more effective than RSWT in the treatment of recalcitrant plantar fasciitis.

In our opinion, FSWT may not always outperform RSWT. According to the results of our experiment, both shock wave therapies are worth considering in the treatment of tennis elbow (the conclusion is consistent with that formulated by Van der Worp et al. (2014) with respect to patellar tendinopathy).

Tennis elbow is a condition caused by strenuous overuse of the muscles. Some patients are unable to perform their duties at work and affected athletes are frequently incapable of participating in training and/or competitions. Sport activities carry much greater risk of some structures of the motor system (e.g. muscle attachments or tendons) being overused, which provides a valid argument for shock wave treatment being used in sport more often than it is now.

Neither of the therapies was observed in the course of treatment to have serious adverse effects. Only 19 patients in the FSWT group and 23 patients in the RSWT group reported feeling pain during procedures, which was gone after the session was over. In 3 patients in the FSWT group and 4 patients in the RSWT group, petechiae and minor edema appeared near the lateral epicondyle while the shock wave was being applied, but they receded before the next session.

The greatest limitation of the study seems to be the absence of two placebo groups receiving a sham focused shock wave and a sham radial shock wave, respectively. In this case, however, creating placebo groups is difficult, as people generally know that shock wave therapy elicits strong physical sensations. Another limitation is that for the lack of a control group the results of treatment with both types of shock waves we obtained cannot be compared with the results of other conservative therapy, one of those that are usually employed to treat tennis elbow, e.g. ultrasound therapy. The number of patients is also relatively small and the long-term effects of the therapies are not presented, however, it is worth noting that the individuals enrolled in our study were generally healthy and did not have any other conditions but tennis elbow. Many of them, particularly those who did not feel pain after the period of treatment or in whom its intensity was considerably reduced, did not show up for follow-up examination 6 and 12 months afterwards. For this reason, representative groups could not be formed.

## Conclusions

Focused and radial shock waves comparably and gradually reduce pain in tennis elbow patients. With subsiding pain, strength of the affected extremity improves.

Neither focused nor radial shock waves improve the function of affected tissues quickly, but they apparently initiate a chain reaction restoring physiological function to affected structures.

## Figures and Tables

**Table 1:** VAS for rest pain, night pain and pain during activity in both groups (X̄±SD)

	Baseline	1 week	3 weeks	6 weeks	12 weeks
Rest pain					
FSWT	2.48±2.06	1.62±1.7*	1.04±1.44***	0.66±1.33***	0.4±1.12***
RSWT	2±2.16	1.64±1.78	1±1.47*	0.38±0.83***	0.26±0.63***
Night pain					
FSWT	1.76±2.28	1.26±1.8	0.84±1.43**	0.6±1.32***	0.4±1.12***
RSWT	1.4±2.06	0.88±1.56	0.48±1.08**	0.2±0.58***	0.2±0.58***
Pain during activity					
FSWT	6.6±1.76	4.5±1.95***	2.98±2.02***	1.96±2.26***	1.6±2.45***
RSWT	6.48±2.06	5.32±2.46***	4.12±2.36***	2.78±2.26***	2.06±2.19***

The post-hoc Tukey’s t-test against the baseline.

* p<0.05,

** p<0.01,

*** p<0.001.

**Table 2:** Change in muscle strength parameters in both groups (X̄±SD)

	Baseline	1 week	3 weeks	6 weeks	12 weeks
Strength of wrist extensors in FSWT group					
Affected [kG]	8.12±3.81	10.40±4.21***	10.88±4.21***	10.96±4.29***	10.75±3.91***
Unaffected [kG]	11.92±4.38	12.88±4.54	12.36±4.23	11.48±4.12	12.08±4.18
Ratio	0.72±0.28	0.84±0.27	0.90±0.24***	0.98±0.23***	0.90±0.28***

Strength of wrist flexors in FSWT group					
Affected [kG]	12.08±4.29	15.20±6.19***	15.68±6.30***	16.60±7.53***	16.80±7.56***
Unaffected [kG]	14.88±6.78	15.48±6.75	15.76±6.95**	15.84±6.93**	15.52±6.74
Ratio	0.93±0.40	1.06±0.39*	1.07±0.31*	1.09±0.32**	1.12±0.30***

Grip strength in FSWT group					
Affected [kG]	36.80±11.53	39.64±11.92**	40.52±11.13***	42.80±11.47***	43.08±11.37***
Unaffected [kG]	40.28±8.91	40.56±8.84	41.16±9.25	41.60±9.10**	41.60±9.04**
Ratio	0.91±0.20	0.97±0.18	0.98±0.14*	1.02±0.11***	1.03±0.12***

Strength of wrist extensors in RSWT group					
Affected [kG]	9.56±4.26	10.84±4.26*	12.04±3.91***	13.12±4.23***	14.04±4.61***
Unaffected [kG]	12.64±3.85	12.68±3.97	13.24±4.01	13.48±4.36	13.00±4.26
Ratio	0.77±0.27	0.86±0.22	0.92±0.19***	1.00±0.23***	1.10±0.19***

Strength of wrist flexors in RSWT group					
Affected [kG]	14.04±4.08	15.32±4.18***	17.36±5.27***	17.40±4.93***	18.36±5.23***
Unaffected [kG]	15.92±4.81	16.16±4.66	17.12±5.09***	16.80±4.76*	17.16±4.54***
Ratio	0.91±0.20	0.98±0.19	1.04±0.19**	1.06±0.18***	1.08±0.17***

Grip strength in RSWT group					
Affected [kG]	38.92±11.65	40.50±11.41	42.08±10.96**	42.80±10.92***	44.48±11.73***
Unaffected [kG]	42.04±11.60	42.36±11.76	42.80±11.61	42.92±11.81	41.68±11.52
Ratio	0.93±0.18	0.97±0.17	1.00±0.14	1.01±0.14**	1.08±0.14***

The post-hoc Tukey’s t-test against the baseline.

* p<0.05,

** p<0.01,

*** p<0.001.

**Table 3:** Percentage change in the analysed parameters in both groups

	FSWT (%±SD)	RSWT (%±SD)	*p*
Rest pain	57±48.69	38.4±55.15	>0.05
Night pain	29.2±44.53	30.33±43.89	>0.05
Pain during activity	74.21±37.79	68.5±29.82	>0.05
Grip strength of the affected extremity	20.45±20.97	17.55±22.6	>0.05
Grip strength of the unaffected extremity	3.43±4.88	−0.78±4.54	>0.05
Wrist extensor strength of the affected extremity	50.31±54.8	61.64±56.27	>0.05
Wrist extensor strength of the unaffected extremity	3.49±20.31	2.61±9.17	>0.05
Wrist flexor strength of the affected extremity	40.61±41.04	33.86±27.5	>0.05
Wrist flexor strength of the unaffected extremity	7.87±19.94	10.33±17.15	<0.05
Extensor strength ratio (affected to unaffected)	43.46±66.95	58.86±56.69	>0.05
Flexor strength ratio (affected to unaffected)	35.64±54.17	23.03±28.28	>0.05
Grip strength ratio (affected to unaffected)	17.3±22.91	18.62±23.01	>0.05

The Mann-Whitney U-test

## References

[b1-jhk-47-127] Alizadehkhaiyat O, Fisher AC, Kemp GJ, Frostick SP (2007). Strength and fatigability of selected muscles in upper limb: Assessing muscle imbalance relevant to tennis elbow. J Elec Kinesiol.

[b2-jhk-47-127] Avancini-Dobrovic V, Frlan-Vrgoc L, Stamenkovic D, Pavlovic I, Schnurrer-Luke Vrbanic T (2011). Radial extracorporeal shock wave therapy in the treatment of shoulder calcific tendinitis. Coll Antropol.

[b3-jhk-47-127] Benjamin SJ, Williams DA, Kalbfleisch JH, Gorman PW, Panus PC (1999). Normalized Forces and Active Range of Motion in Unilateral Radial Epicondylagia (Tennis Elbow). J Orthop Sports Phys Ther.

[b4-jhk-47-127] Cacchio A, Paoloni M, Barile A, Don R, de Paulis F, Calvisi V, Ranavolo A, Frascarelli M, Santilli V, Spacca G (2006). Effectiveness of radial shock-wave therapy for calcific tendinitis of the shoulder: single-blind, randomized clinical study. Phys Ther.

[b5-jhk-47-127] Gerdesmeyer L, Frey C, Vester J, Maier M, Weil L, Weil L, Russlies M, Stienstra J, Scurran B, Fedder K, Diehl P, Lohrer H, Henne M, Gollwitzer H (2008). Radial extracorporeal shock wave therapy is safe and effective in the treatment of chronic recalcitrant plantar fasciitis: results of a confirmatory randomized placebo-controlled multicenter study. Am J Sport Med.

[b6-jhk-47-127] Gunduz R, Malas FU, Borman P, Kocaoglu S, Ozcakar L (2012). Physical therapy, corticosteroid injection, and extracorporeal shock wave treatment in lateral epicondylitis: Clinical and ultrasonographical comparison. Clin Rheumatol.

[b7-jhk-47-127] Haake M, König IR, Decker T, Riedel C, Buch M, Müller HH (2002). Extracorporeal shock wave therapy in the treatment of lateral epicondylitis: a randomized multicenter trial. J Bone Joint Surg Am.

[b8-jhk-47-127] Hammer DS, Rupp S, Ensslin S, Kohn D, Seil R (2000). Extracorporal shock wave therapy in patients with tennis elbow and painful heel. Arch Orthop Trauma Surg.

[b9-jhk-47-127] Ibrahim MI, Donatelli RA, Schmitz C, Hellman M, Buxbaum F (2010). Chronic plantar fasciitis treated with two session of radial extracorporeal shock wave therapy. Foot Ankle Surg.

[b10-jhk-47-127] Johnson GW, Cadwallader K, Scheffel SB, Epperly TD (2007). Treatment of lateral epicondylitis. Am Fam Physician.

[b11-jhk-47-127] Ko JY, Chen HS, Chen LM (2001). Treatment of Lateral Epicondylitis of the Elbow With Shock Waves. Clin Orthop Relat Res.

[b12-jhk-47-127] Krischek O, Hopf C, Nafe B, Rompe JD (1999). Shock-wave therapy for tennis and golfer’s elbow - 1 year follow-up. Arch Orthop Trauma Surg.

[b13-jhk-47-127] Lee SS, Kang S, Park NK, Lee CW, Song HS, Sohn MK, Cho KH, Kim JH (2012). Effectiveness of initial extracorporeal shock wave therapy on the newly diagnosed lateral or medial epicondylitis. Ann Rehabil Med.

[b14-jhk-47-127] Lohrer H, Nauck T, Dorn-Lange NV, Scholl J, Vester JC (2010). Comparison of radial versus focused extracorporeal shock waves in plantar fasciitis using functional measures. Foot Ankle Int.

[b15-jhk-47-127] Melegati G, Tornese D, Bandi M, Rubini M (2004). Comparison of two ultrasonographic localization techniques for the treatment of lateral epicondylitis with extracorporeal shock wave therapy: a randomized study. Clin Rehabil.

[b16-jhk-47-127] Ogden JA, Toth-Kischkat A, Schultheiss R (2001). Principles of shock wave therapy. Clin Orthop Relat Res.

[b17-jhk-47-127] Oken O, Kahraman Y, Ayhan F, Canpolat S, Yorgancioglu ZR, Oken OF (2008). The short-term efficacy of laser, brace, and ultrasound treatment in lateral epicondylitis: a prospective, randomized, controlled trial. J Hand Ther.

[b18-jhk-47-127] Rompe JD, Hopf C, Kullmer K, Heine J, Burger R (1996). Analgesic effect of extracorporeal shock-wave therapy on chronic tennis elbow. J Bone Joint Surg Br.

[b19-jhk-47-127] Shiri R, Viikari-Juntura E (2011). Lateral and medial epicondylitis: Role of occupational factors. Best Pract Res Clin Rheumatol.

[b20-jhk-47-127] Snijders CJ, Volkers AC, Mechelse K, Vleeming A (1987). Provocation of epicondylagia lateralis (tennis elbow) by power grip or pinching. Med Sci Sports Exerc.

[b21-jhk-47-127] Spacca G, Necozione S, Cacchio A (2005). Radial shock wave therapy for lateral epicondylitis: a prospective randomised controlled single-blind study. Eur Med Phys.

[b22-jhk-47-127] Speed CA, Nichols D, Richards C, Humphreys H, Wies JT, Burnet S, Hazleman BL (2002). Extracorporeal shock wave therapy for lateral epicondylitis - a double blind randomised controlled study. J Orthop Res.

[b23-jhk-47-127] Stasinopoulos D, Johnson MI (2004). Cyriax physiotherapy for tennis elbow/lateral epicondylitis. Br J Sports Med.

[b24-jhk-47-127] Stasinopoulos D, Stasinopoulou K, Johnson MI (2005). An exercise programme for the management of lateral elbow tendinopathy. Br J Sports Med.

[b25-jhk-47-127] Thiel M (2001). Application of shock waves in medicine. Clin Orthop Relat Res.

[b26-jhk-47-127] Trinh KV, Phillips SD, Ho E, Damsma K (2004). Acupuncture for the allevi-ation of lateral epicondyle pain: a systematic review. Rheumatology.

[b27-jhk-47-127] Trudel D, Duley J, Zastrow I, Kerr EW, Davidson R, MacDermid JC (2004). Rehabilitation for Patients with Lateral Epicondylitis: A Systematic Review. J Hend Ther.

[b28-jhk-47-127] Van der Worp H, Zwerver J, Hamstra M, van den Akker-Scheek I, Diercks RL (2014). No difference in effectiveness between focused and radial shockwave therapy for treating patellar tendinopathy: a randomized controlled trial. Knee Surg Sports Traumatol Arthrosc.

